# Virulence analysis of *Staphylococcus aureus* in a rabbit model of infected full-thickness wound under negative pressure wound therapy

**DOI:** 10.1007/s10482-017-0938-z

**Published:** 2017-09-11

**Authors:** Daohong Liu, Zhirui Li, Guoqi Wang, Tongtong Li, Lihai Zhang, Peifu Tang

**Affiliations:** 1 Department of Orthopedics, The 309th Hospital of PLA, Beijing, 100091 China; 2 Department of Orthopedics, The General Hospital of People’s Liberation Army, Beijing, 100853 China; 3 Department of Orthopedics, Chinese PLA General Hospital and Hainan Branch, Sanya, 572013 China; 4 Department of Orthopedics, Tianjin Hospital, Tianjin, 300211 China

**Keywords:** Bacterial virulence, Infection, Gene expression, Negative pressure wound therapy, Bioluminescent imaging

## Abstract

The aim of this study was to evaluate the virulence of *Staphylococcus aureus* in a controlled animal study using the standard sterile gauze and negative pressure wound therapy (NPWT), including activation of agr, gene expression and production of virulence foctors and depth of bacterial invasion. The tissue specimens were harvested on days 0 (6 h after bacterial inoculation), 2, 4, 6, and 8 at the center of wound beds. Laser scanning confocal microscopy was performed to obtain bioluminescent images which were used to measure the depth of bacterial invasion. The *agrA* expression of *S.aureus* and the transcription and production of virulence factors including Eap, Spa and α-toxin were significantly different. The bacterial invasion depth was significantly less with effect of NPWT. The markedly different activation of quorum sensing systems that enable cell-to-cell communication and regulation of numerous colonization and virulence factors result in distinct gene expression and pathogenicity over time in different microenvironment. Thus, the agr system represents a fundamental regulatory paradigm that can encompass different adaptive strategies and accommodate horizontally acquired virulence determinants.

## Introduction

Wound infection is a serious complication that acts to prolong healing and extends human suffering. *Staphylococcus aureus* is the predominant pathogen that typically causes soft tissue infections (Moet et al. 2007; Lowy 1998). Following wounding, the local microenvironment in a wound can provide favourable conditions for *S. aureus* to colonise and proliferate, and secrete toxins, to spread further infection, which hinders the infected wound healing (Davies et al. 2007). Therefore, preventing infection has been a basic principle for wound care. In the last two decades, negative pressure wound therapy (NPWT) has been widely used for the treatment of various wounds (Fleischmann et al. 1993; Morykwas and Argenta 1997; Mooney et al. 2000; Song et al. 2003) and has shown significant clinical benefits in infected wound healing (Morykwas and Argenta 1997; Fleischmann et al. 1998; Pinocy et al. 2003; Fleck et al. 2002). Unlike traditional therapies for wound care, such as open wound management, which lead to a decrease of *S. aureus* infection, several studies found that *S. aureus* showed a significant increase in wounds that continued to show gross and microscopic improvement when treated with standard NPWT (Weed et al. 2004; Moues et al. 2004; Boone et al. 2010; Lalliss et al. 2010).

The pathogenicity of *S. aureus* includes several steps, such as invasion of the host, adherence to and persistence in tissues, and escape from the immune system, which involving the coordinated expression of diverse virulence factors in response to environmental cues. The accessory gene regulator (agr) is a quorum sensing system that controls expression of a substantial proportion of the virulence genes of *S. aureus* (Dunman et al. 2001; Booth et al. 1995) and plays a central role in staphylococcal pathogenesis. Extracellular adherence protein (Eap) enhances the adhesion of staphylococci to the target tissue by binding to a variety of extracellular matrix components, such as vitronectin, fibrinogen, fibronectin and collagens (Kreikemeyer et al. 2002; McGavin et al. 1993; Hansen et al. 2006). An unusual property of Eap is its ability to bind back to the cell surface and aggregate *S. aureus* through Eap–Eap interactions (Palma et al. 1999). Eap also reduces neutrophil recruitment and diminishes leukocyte adhesion to endothelial cells (Haggar et al. 2004; Chavakis et al. 2002). Staphylococcal protein A (Spa) on the microbial surface inhibits antibody-mediated phagocytosis by blocking the Fc portion of IgG (Foster and McDevitt 1994; Uhlen et al. 1984). *S. aureus* α-toxin, encoded by *hla*, is one of a major extracellular virulence factors. α-toxin targets a broad range of host cell types as a cytotoxin and plays an essential role in pulmonary, intraperitoneal and inframammary infections (Walev et al. 1993; Bubeck Wardenburg et al. 2007). The effects of α-toxin include cell lysis, release of proinflammatory mediators and cytokines, and induction of apoptosis.

NPWT as a kind of physiotherapeutic can change the microenvironment of microorganisms. Therefore, by analysing temporal expression and the production of virulence factors in an animal model, we aimed to test the hypothesis that the NPWT-mediated inhibition of global regulators might reduce the virulence of *S. aureus*. The main aim of this study was to evaluate the activation of agr, investigate the expression and production of virulence factors, and measure the depth of bacterial invasion in a controlled animal study using standard sterile gauze and NPWT.

## Materials and methods

### Animals

All animal work was performed in accordance with protocols approved by the Medical Ethics Committee of the Chinese PLA General Hospital. Young, adult female Japanese white rabbits (specific pathogen free, aged 3–6 months, approximately 3 kg) were acclimated to standard housing and feed. All animals were housed in individual special cages under constant temperature (22 °C) and humidity (45%) with a 12-h light–dark cycle. A total of 68 animals were used for this study.

### Bacterial preparation and inoculation

*Staphylococcus aureus* (RN6390-GFP), with constitutive expression of the green fluorescent protein (GFP), was obtained from the Chinese PLA Institute for Disease Control and Prevention (Beijng, China). Once the wound model was created, the rabbits were inoculated with 0.5 mL of >10^8^ colony forming U/mL *S. aureus*. The wound was bandaged with sterile gauze dressings.

### Wound creation

Three days prior to the experimental procedure, the backs of the animals were shaved with a standard electric shaving machine. 8% sodium sulfide solution (or commercial depilatory cream) was used to obtain a smooth and hairless skin. All animals were anaesthetised intramuscularly injection with Ketamine (50 mg/kg) and Xylazine (5 mg/kg) before surgical procedures. Following intradermal injection of 1% lidocaine, bilaterally symmetrical standardised 2.5 cm-diameter full-thickness circular segments were excised beside the spine in the middle of back, down to the deep fascia, from the back skin prepared with povidone iodine solution, and a wound area of approximately 5 cm^2^ was created for each side. The total wound area was less than 10% of the animal’s total body surface area.

### Treatment and wounds harvesting

Respectively, bilateral wounds were covered with a poly(vinyl alcohol) shrink formaldehyde bubble dressing (VSD Medical Science and Technology Co., Ltd., Wuhan, China) as the negative-pressure wound therapy (NPWT) group or sterile gauze dressing as the control randomly. The NPWT devices were all set to continuous suction at a negative pressure of 125 mmHg. Gauze dressings were checked daily and the NPWT dressings changed every 48 h after bulb syringe irrigation of the wounds in all groups.

Wound data were collected as described by Morykwas and Argenta (1997) at day 0 (6 h after bacterial inoculation) and days 2, 4, 6 and 8. The animals were anesthetised and prepared for surgery. Having removed the exudates on the surface of the defect with sterile saline solution, biopsies were taken at the center of wound beds under aseptic conditions using a scalpel and stored in asepsis centrifugal tubes at 4 °C.

For the quantitative real time polymerase chain reaction (RT-PCR) and Western blot analyses carried out on days 0, 2, 4, 6 and 8 in wounds to determine the temporal expression and production of virulence factors in vivo, wounds were harvested as described (Gurjala et al. 2011) and samples stored at −80 °C until the time of analysis.

**Fig. 1:**
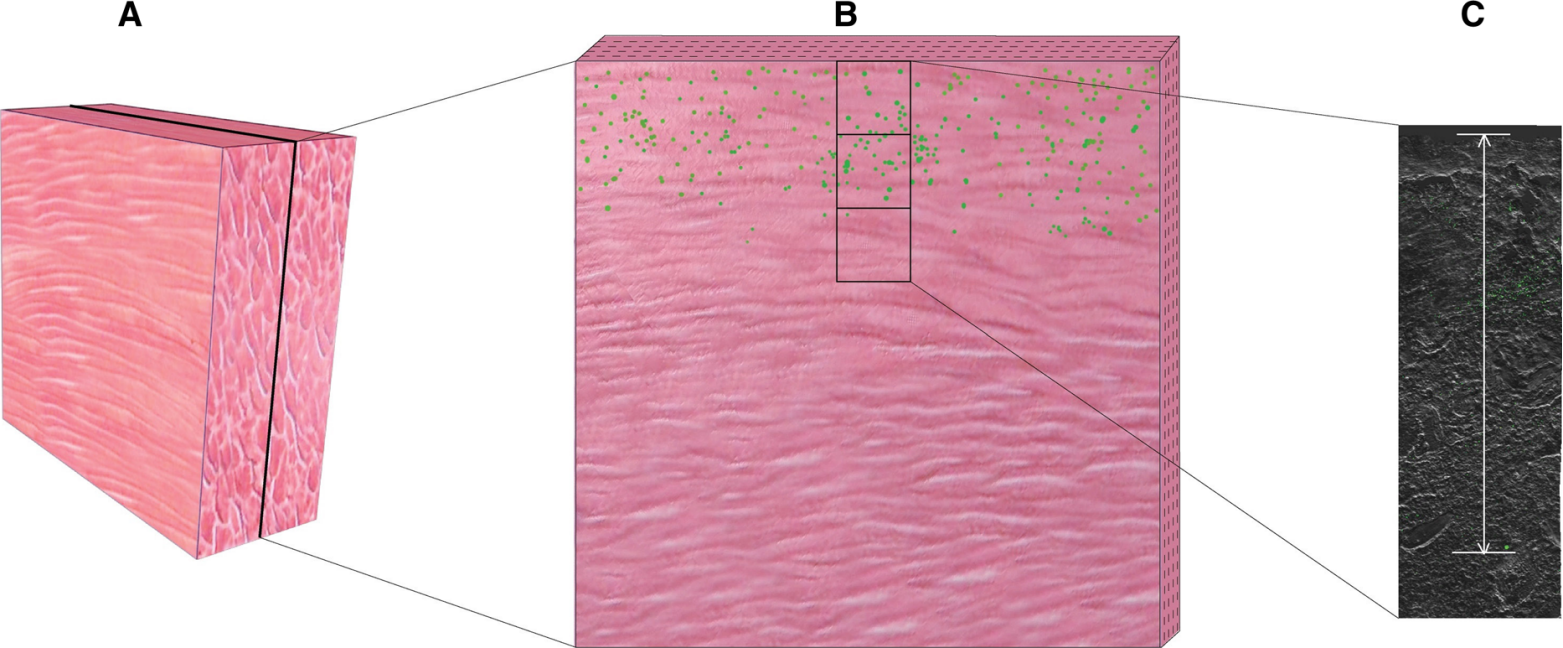
Muscle specimen sampling for microscopy. The tissue specimens were harvested from erector spinae muscle at the center of wound beds. The samples and observation setup is represented. **a** The muscle specimens were longitudinally excised as 1 cm × 0.5 cm × 1 cm cubes perpendicular to the surface of the wound. **b** The specimen was sectioned with 6 µm thickness, and three views were created continuously along the *Y*-axis perpendicular to the surface of the wound. **c** The invasion depth of GFP-labeled *S. aureus* was measured from tissue boundary to the deepest location

For fluorescent imaging by laser scanning confocal microscopy, animals were sacrificed on days 0 (6 h after bacterial inoculation), 2, 4, 6 and 8. At the time of euthanasia, the tissue specimens were harvested from the erector spinae muscle at the center of wound beds. The specimens were longitudinally excised 1 cm × 0.5 cm × 1 cm cubes perpendicular to the surface of the wound and embedded in OCT compound, snap frozen in liquid nitrogen and stored at −80 °C until cryosectioning (Fig. 1a).

### Total mRNA extraction and RT-PCR analysis

300 mg of tissue specimens was removed from the erector spinae of the animals. Using Trizol (Invitrogen) according to the manufacturer’s protocol, the supernatant was harvested for RNA extraction; the pellet was resuspended in 250 μg/mL lysostaphin (OMEGA), incubated at 37 °C for 15 min, then used for the bacterial RNA extraction. RNA was reverse transcribed into cDNA using the TIANScript RT Kit (TIANGEN). For quantitative analysis of the expression level of mRNAs, real time quantitative PCR analyses using SYBR FAST qPCR Kit Master Mix (2×) Universal (KAPA) were performed utilising an ABI7900HT sequence detection system (ABI). Cycling conditions were as follows: one cycle at 95 °C for 3 min; 40 cycles at 95 °C for 3 s, 60 °C for 20 s; and one dissociation step at 95 °C for 15 s, 60 °C for 15 s, and 95 °C for 15 s.

Expression of the bacterial genes were normalised against 16S rRNA expression, to get Δ*C*_t_. All samples were analysed in triplicate and the $2^{{ - \Delta \Delta C_{\text{t}} }}$ method was used to calculate gene expression. The results were expressed as the fold change of the different genes compared with the housekeeping gene.

### Western blot analysis

The tissue specimens harvested above were homogenised and incubated on ice for 30 min in the presence of RadioImmuno Precipitation Assay (RIPA) buffer. Supernatants were collected by centrifugation at 13000 rpm for 30 min (4 °C). 25 μL of supernatant was loaded on 8% SDS-PAGE gels. Western Blot analysis for the detection of α-toxin, Eap and Spa in wound extracts was performed as previously described (Qiu et al. 2010). Antibodies for detection of Eap, Spa and α-toxin were purchased from Abcam.

### Laser scanning confocal microscopy and fluorescent quantitative

The muscle specimens were cut into 6 μm thick sections with the use of a cryostat and mounted on glass slides for viewing using fluorescent microscopy (Fig. 1b). Section slides were observed with an argon confocal laser scanning microscope (Olympus FV1000, Tokyo, Japan) to capture the invasion depth of the GFP-labeled *S. aureus*. Perpendicular to the surface of the wound, the images of three views were continuously created along the y-axis (Fig. 1c) according to the results of a pilot trial. The tissue boundary was identified by referring to differential interference contrast (DIC) images. The scan speed was set at 4 ms/pixel. The scan area was 512 × 512 pixels, and the power of the 488 nm laser was set at 4.5% according to the power slider in the FV1000 microscope. To determine the invasion depth of GFP-labeled *S. aureus* from the tissue boundary to the deepest location, digital images were captured with the same parameters and measured by two blinded independent observers. The depths of the bacterial fluorescent signal from samples was quantified by FV10-ASW 4.1 software embedded on Olympus FV-1000.

### Statistical analysis

All data were presented as the mean ± standard deviation, and serial changes of virulence gene expression and western blot analysis were compared using a two-way analysis of variance (ANOVA) with repeated measures, followed by a paired design multivariate analysis of variance to test multiple pairwise comparisons. Bonferroni Significant Difference tests were performed on within-subject characteristics changes over time. Data of bacterial invasion depth were assessed with a paired design multivariate analysis of variance between two groups, and one-way ANOVA within each group. All statistical analyses were performed with SPSS software (SPSS 19.0, SPSS Inc., Chicago, IL, USA). The significance level was set at P < 0.05.

## Results

Seventy-eight rabbits (88%) survived until the scheduled experimental plan was completed after bacterial inoculation. All of the wounds presented macroscopic signs of infection, such as necrosis and exudates, and harboured *S. aureus* after 54 h at autopsy.

### Gene expression

**Fig. 2:**
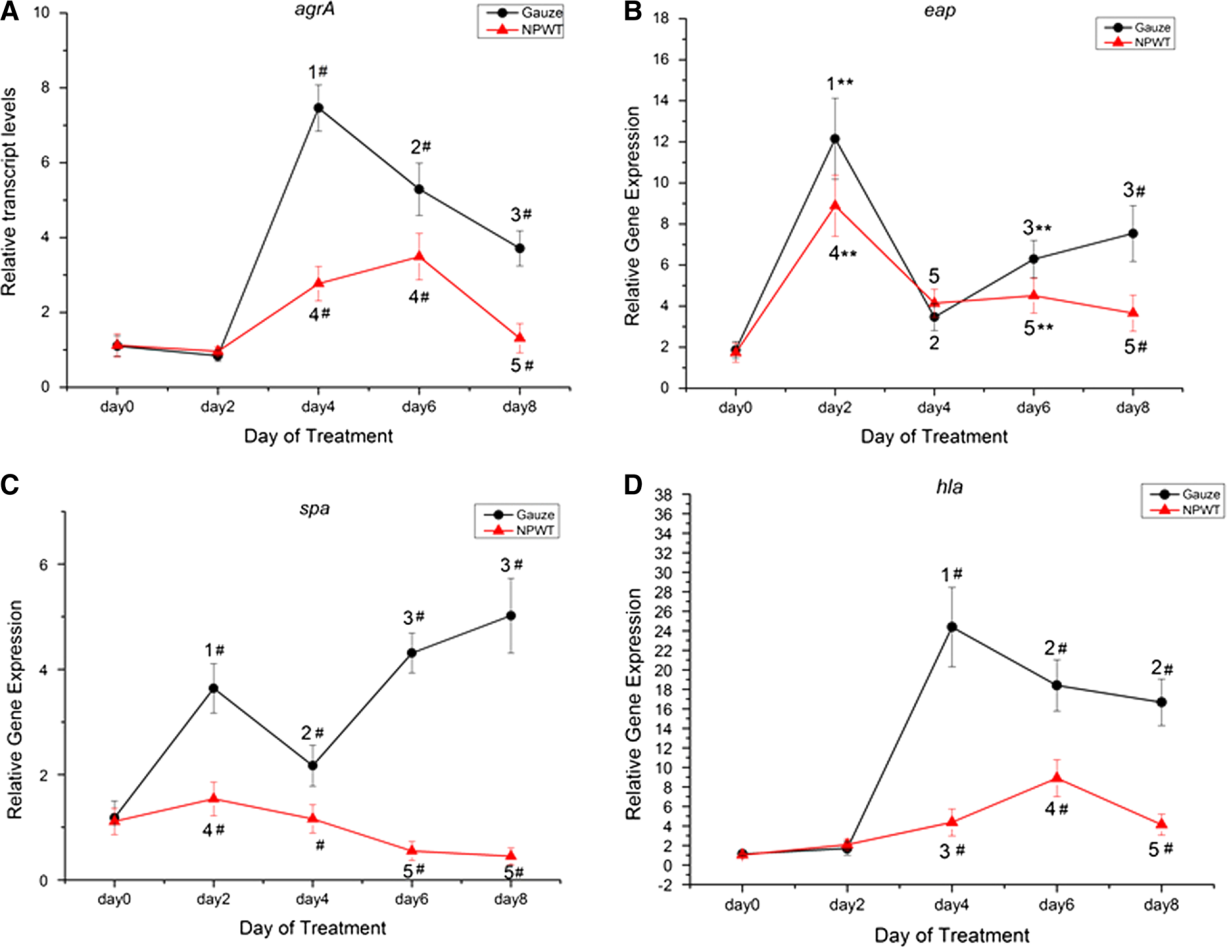
Differences in gene expression in the two treatment groups over time. **a** The difference of *agrA* expression was significant between the groups at day 4, 6 and day 8 (^#^P < 0.001). In the sterile gauze dressing group, *agrA* expression significantly increased at day 4 (^1^P < 0.001 vs. day 0 and day 2) and decreased at day 6 and 8 (^2^P < 0.01 vs. day 0, 2 and day 4; ^3^P < 0.01 vs. day 0, 2, 4 and day 6). Gene expression significantly increased at day 4 and day 6 (^4^P < 0.001 vs. day 0 and day 2), and significantly decreased at day 8 compared with day 4 and day 6 (^5^P < 0.01). **b** The expression of *eap* was significantly different in the NPWT group compared with the sterile gauze dressing group at days 2 and 6 (**P < 0.01), and at day 8 (^#^P < 0.001). In the sterile gauze dressing group, significant differences were shown at day 2 (^1^P < 0.001 vs. day 0), and at day 4 (^2^P < 0.001 vs. day 0 and day 2), and at day 6 and day 8 (^3^P < 0.05 vs. days 0, 2 and 4). In the NPWT group, expression of *eap* significantly increased at day 2 (^4^P < 0.001 vs. day 0), and decreased at days 4, 6 and 8 (^5^P < 0.01 vs. day 0 and day 2). **c** The expression of *spa* was significantly different between groups at days 2, 4, 6 and 8 (^#^P < 0.001). Significant differences were shown at day 2 (^1^P < 0.001 vs. day 0), at day 4 (^2^P < 0.001 vs. day 0 and day 2) and at days 6 and 8 (^3^P < 0.05 vs. days 0, 2, and 4) in the sterile gauze dressing group. In the NPWT group, the expression of *spa* increased at day 2 (^4^P < 0.05 vs. day 0), and significantly decreased at days 6 and 8 (^5^P < 0.01 vs. days 0, 2 and 4). **d** The expression of *hla* was significantly different between groups at days 4, 6 and 8 (^#^P < 0.001). In the sterile gauze dressing group, α-toxin significantly increased at days 4, 6 and 8 (^1^P < 0.001 vs. day 0 and day 2; ^2^P < 0.05 vs. days 0, 2 and 4). Gene expression significantly increased at day 4 and day 6 (^3^P < 0.01 vs. day 0 and day 2; ^4^P < 0.01 vs. day 0, 2 and day 4), and significantly decreased at day 8 compared with day 6 (^5^P < 0.01)

The selected gene expression was analysed using real-time PCR. The expression changes over time of *agrA*, *eap*, *hla* and *spa* were all statistically different (P < 0.001) in the sterile gauze dressing and NPWT groups. The expression of *agrA* showed a sharp rise from day 2 to day 4 in the sterile gauze dressing group when compared with day 0 (P < 0.001), and was also significantly higher than in the NPWT group (P < 0.001) at day 4, day 6 and day 8. Significant differences were shown in the NPWT group at day 4 and day 6 compared with day 0 (P < 0.001) (Fig. 2a). A significant increase in *eap* expression was observed at day 2 in both groups (P = 0.001), then decreased at day 4 (P = 0.074); significant differences between the groups were observed at day 6 and day 8(P = 0.003; P < 0.001, respectively). Within each group significant differences were shown from day 2 to day 8 compared with day 0 (P < 0.001) (Fig. 2b). The expression of *spa* significantly (P < 0.001) increased in the sterile gauze dressing group from day 2 to day 8, compared with day 0 (P < 0.001); and in the NPWT group significantly decreased at day 6 and day 8 compared with day 0 (P = 0.002; P = 0.001, respectively) (Fig. 2c). The trend of *hla* expression change over time was similar to *agrA* in the sterile gauze dressing group, and significant difference was shown in the NPWT group from day 4 to day 8 compared with day 0 (P = 0.003; P = 0.001; P < 0.001; P < 0.001, , respectively) (Fig. 2d). The sterile gauze group was significantly different to the NPWT group at days 4–8. Overall, the treatment and time of *agrA*, *eap*, *hla* and *spa* expression changes had an interaction (two-way repeated measured ANOVA).

### Assessment of bacterial virulence

**Fig. 3:**
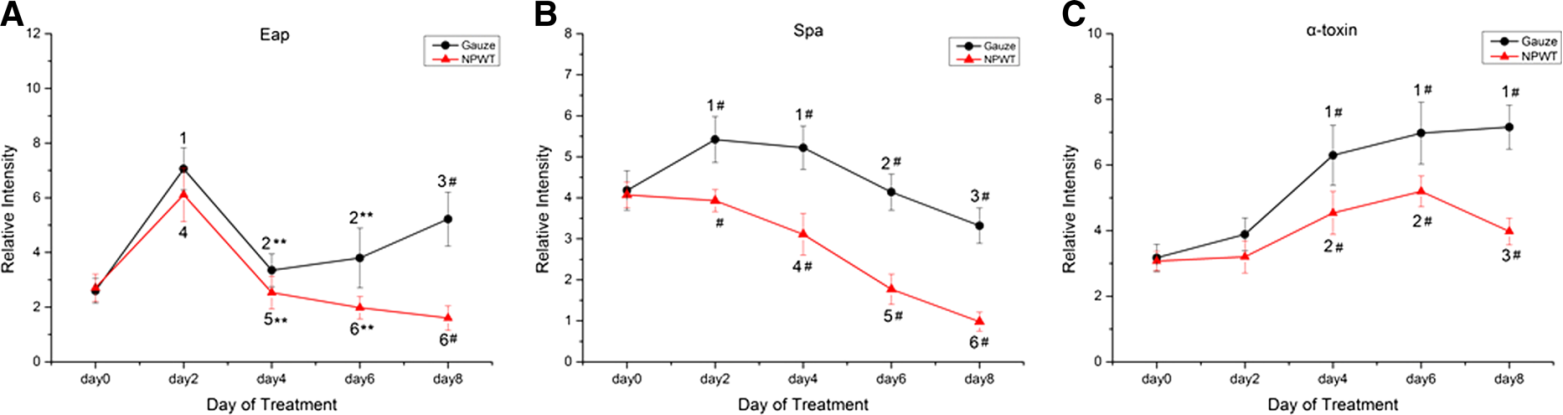
Differences in protein production in the two treatment groups over time determined by Western blotting. **a** The production of Eap was significantly different in the NPWT group compared with the sterile gauze dressing group at day 4, 6 (**P < 0.01) and at day 8 (^#^P < 0.001). Eap significantly increased at day 2 (^1,4^P < 0.001 vs. day 0) in both groups. In the sterile gauze dressing group, significant difference were shown at day 4 and day 6 (^2^P < 0.001 vs. day 2), and at day 8 (^3^P < 0.05 vs. days 0, 2 and 4). **b** Spa was significantly different between groups at days 2, 4, 6 and 8 (^#^P < 0.001). In the sterile gauze dressing group, significant differences were shown at day 2 and day 4 (^1^P < 0.05 vs. day 0), at day 6 (^2^P < 0.01 vs. day 2 and day 4), and at day 8 (^3^P < 0.05 vs. days 0, 2, 4 and 6). In the NPWT group, Spa gradually decreased at day 4, 6 and day 8 (^4^P < 0.05 vs. day 0 and day 2; ^5^P < 0.01 vs. days 0, 2 and 4; ^6^P < 0.01 vs. days 0, 2, 4 and 6). **c** The α-toxin was significantly different between the groups at day 4, 6 and day 8 (^#^P < 0.001). In the sterile gauze dressing group, α-toxin significantly increased at day 4, 6 and day 8 (^1^P < 0.001 vs. day 0 and day 2). In the NPWT group, the α-toxin increased at day 4 and 6 (^2^P < 0.05 vs. day 0 and day 2), and decreased at day 8 compared with day 4 and day 6 (^3^P < 0.01)

Western blot analysis was used to assess the production of Eap, Spa and α-toxin by *S. aureus* that were obtained from the sterile gauze dressing group and NPWT groups. The production over time of Eap, Spa and α-toxin were all statistically different (P < 0.001) in the sterile gauze dressing group and NPWT group. The increase in Eap production was not significantly different between the two groups (P = 0.082) at day 2; within the sterile gauze dressing group a significant increase was shown at day 8 compared with day 0 (P < 0.001), and a significant decrease was shown in the NPWT group at day 6 and day 8 compared with day 0 (P = 0.001; P = 0.016, respectively) (Fig. 3a). The production of Spa was significantly different between the two groups (P < 0.001) from day 2 to day 8; a significant increase was observed at day 2 and day 4 (P = 0.013; P = 0.029, respectively) and significant decrease was observed at day 8 (P = 0.028) compared with day 0 in the sterile gauze dressing group. In the NPWT group, Spa significantly decreased from day 4 to day 8 compared with day 0 (P = 0.025; P < 0.001; P < 0.001, respectively) (Fig. 3b). The α-toxin production significantly increased from day 4 to day 8 compared with day 0 (P < 0.001) in the sterile gauze dressing group, and significant difference was shown from day 4 to day 8 (P < 0.001) between the two groups; in the NPWT group no significant difference was shown at day 8 compared with day 0 (P = 0.194) (Fig. 3c). The treatment and time of Eap, Spa and α-toxin production changes had an interaction (two-way repeated measures ANOVA).

### Bacterial invasion depth

**Fig. 4:**
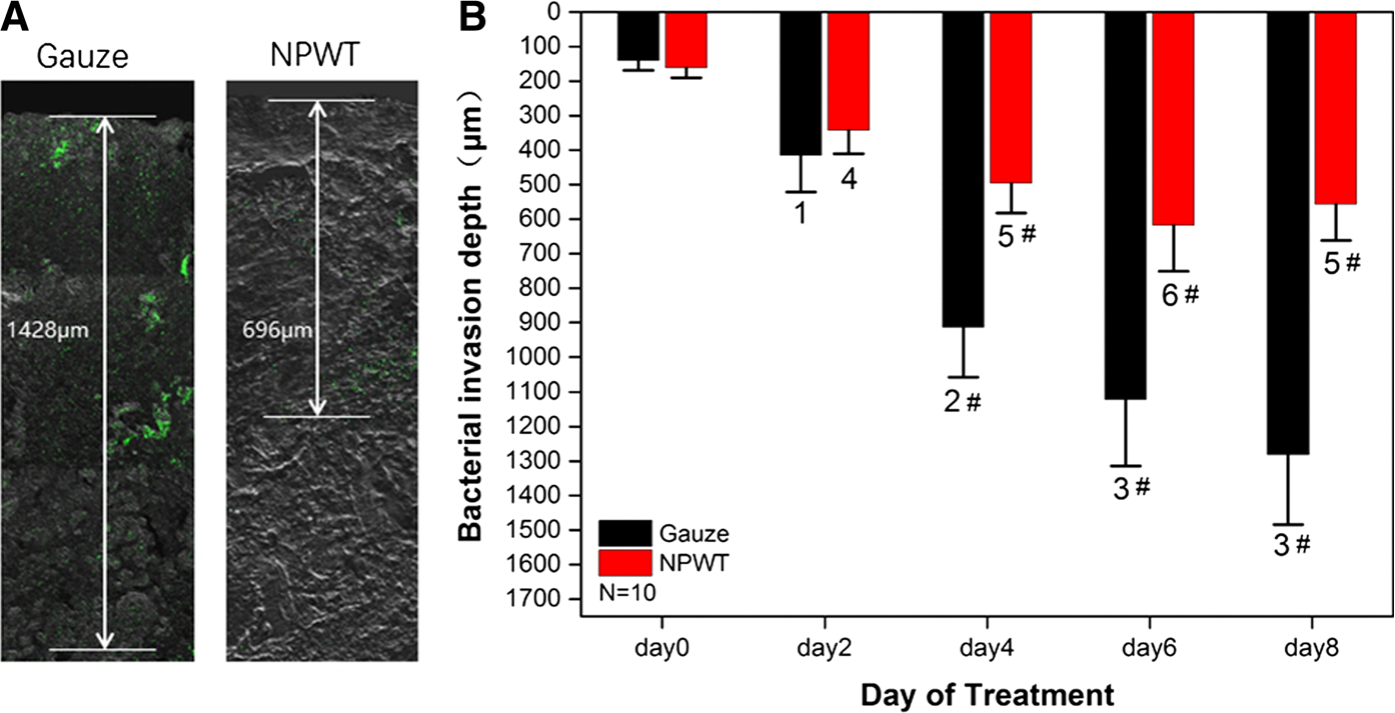
Bacterial invasion depth observed by laser scanning confocal microscopy. **a** This figure shows the depth of infection in Gauze and NPWT on day 8. **b** Bacterial invasion depth significantly descended in NPWT group compared with sterile gauze dressing group at day 4, 6 and day 8 (^#^P < 0.001). Bacterial invasion depth increased at day 2 (^1^P < 0.01 vs. day 0), and at day 4 (^2^P < 0.001 vs. day 0 and day 2), and at day 6 and day 8 (^3^P < 0.05 vs. day 0, 2 and day 4) in sterile gauze dressing group. In NPWT group, the tendency of bacterial invasion depth increase was similar from day 2 to day 6 (^4^P < 0.01 vs. day 0; and ^5^P < 0.001 vs. day 0 and day 2; and ^6^P < 0.05 vs. day 0, 2 and day 4), however, no significant difference was showed at day 6 compared with day 8 (P > 0.05)

Laser scanning confocal microscopy was used to observe bacterial invasion of the GFP-labeled *S. aureus* in tissue (Fig. 4a). Imaging validated the different invasion depth of viable bacteria within wounds over the course of time used in our model. Results of paired design multivariate ANOVA under the significant level of 0.05 indicated statistically significant differences between the two treatment groups (F = 26.195, P < 0.001). The bacterial invasion depth before treatment was similar (p = 0.734). The invasion depth was significantly different between two groups from day 4 to day 8 (P < 0.001). In the sterile gauze dressing group, the mean of invasion depth increased continuously from day 0 to day 8. No significant difference was showed at day 6 compared with day 8 (P = 0.202), and the mean of invasion depth was 1122 ± 192 μm versus 1282 ± 202 μm respectively. In the NPWT group, the mean of invasion depth increased continuously from day 0 to day 6. The mean depth at day 8 was less than at day 6, 557 ± 105 μm versus 618 ± 133 μm respectively. However, the difference was not statistically significant (P = 1) (Fig. 4b).

## Discussion

The results presented here determined that the temporal pattern of gene expression, virulence factor production and invasion depth of *S. aureus* infected full-thickness wound changes over time were all statistically different in the sterile gauze dressing group and the NPWT group. The effect of NPWT by continuous negative pressure suction changed the environment that enables bacteria to invade the target tissue and maintain the infection. The decline in activity of the agr quorum sensing system and the decrease of virulence factor expression were shown using an in vivo model treated with NPWT. Lower amounts of α-toxin and the cell surface virulence factors (Eap and Spa) were found in the NPWT group compared with sterile gauze group by western blot. Bacterial spread and invasion was more pronounced within wound tissue, where the invasion depth in wounds treated with sterile gauze was over two times higher than that seen in wounds treated with NPWT (1282 ± 202 μm vs. 557 ± 105 μm).

The sequence of *agrA* in the *agr* locus is conserved across the four *S. aureus* groups (George and Muir 2007). AgrA binds to both the P2 and P3 promoters to initiate *agr* transcription. Increase in *agrA* transcription activates the agr system. Entry of a relatively large number of bacteria into the wound tissue of the rabbit leads to late activation of the agr quorum sensing system at day 4 in the sterile gauze group and at day 6 in the NPWT group. In fact, in vivo agr may undergo even more complex activation patterns, given that it has been shown to be activated in an eclipse-type manner with very late activation (Wright et al. 2005). We suggest that the bacteria have not established large populations early in infection, and cannot trigger *agr* autoinduction activation, which relies on increasing *S. aureus* densities. In addition, the expression of *agr* is modified after interactions with host cells. Rothfork et al. (2004) documented the capacity of neutrophils to attenuate *agr* expression by the generation of reactive oxygen and nitrogen intermediates. Our results showed that transcription from the *agrA* locus was significantly decreased when *S.aureus* was grown in the NPWT group. We have observed that bacteria on the surface of wounds and within the tissue only formed discrete single colonies when treated with NPWT, and differed from the numerous bacteria that accumulate to form localised large colonies in the sterile gauze group (unpublished data). We also note that NPWT can effectively promote neutrophil accumulation in the early period of infection, especially in the shallow wound bed (unpublished data). It is likely that both effects can inhibit the *agr* expression.

We have observed that there is a significant increase in the expression of *eap* and *spa* on day 2 in the sterile gauze group. However, on day 4 the expression of *eap* and *spa* decreased. The expression levels of *eap* and *spa* are negatively controlled by *agr* (Dunman et al. 2001; Huntzinger et al. 2005), so *agr* activation may lead to the reduction in the expression and production of these two virulence factors. Following the agr system gradually becoming inactive, the expression and production of the two virulence factors increases, which should improve bacterial adhesion and colonisation to other sites in infected tissue. However, the expression of *eap* and *spa* were significantly lower in the NPWT group in comparison to the sterile gauze group. One reason for the difference in *eap* and *spa* transcription is NPWT might lead to the activation of differing regulatory circuits. Another reason might be that the cell surface proteins Eap and Spa are depleted by enhancing the local immune responses when NPWT is performed.

Like most staphylococcal extracellular proteins, α-toxin is not expressed constitutively but is centrally regulated by the agr quorum sensing system. *agr* activates *hla*, which encodes the α-toxin, at both the transcriptional and translational levels (Novick 2003). *S. aureus* produces exotoxins via agr quorum sensing signaling which allow *S. aureus* to spread from the colonisation sites to the deeper tissue. The gradual increase in α-toxin following the reduction of *agr* in the sterile gauze group (Fig. 3c) may be caused by the accumulation of α-toxin in tissue, but the active drainage during NPWT may results in the significant decrease of α-toxin.

Bacteria within a wound can range from contamination, colonization, localised infection, spreading infection and ultimately to systemic infection if not appropriately controlled (Lindstedt et al. 2012). The above different states describe the dynamic process of bacteria wound invasion. Confocal imaging of *S. aureus* infection using bioluminescent engineered bacterial strains enables the assessment the bacterial invasion depth which can reflect the invasion process. We found that the bacterial invasion depth over time showed distinct differences between the two groups with different treatments in our study. Such significant difference in invasion depth is in accordance with the different bacterial gene expression and production of virulence factors, but does not corelate with the bacterial count. Therefore, our study suggests NPWT may play a role by regulating the expression of virulence factors to prevent *S. aureus* from invading further, even though it does not obviously change the amount of bacteria present.

Our results show that regulation of bacterial gene expression and pathogenicity over time in an vivo model differ with each form of treatment, even though the amount of bacteria is similar. The methods of our study provide a direct approach for the evaluation of putative virulence factors involved in *S. aureus* infection of full-thickness wounds in animals. NPWT may change the microenvironment of the microorganisms, and lead to the differences in the activation of the *agr* quorum sensing system, which results in distinct gene expression and pathogenicity over time. The agr quorum sensing system, as the fundamental regulator of *S. aureus*, can determine the expression profile of virulence determinants. The expression of various virulence factors changes the microenvironment, provokes cell-to-cell communication, influences regulation of colonisation factors and which then affects the development of infection.

## References

[CR1] Boone D, Braitman E, Gentics C, Afthinos J, Latif J, Sordillo E, Todd G, Lantis JC (2010). Bacterial burden and wound outcomes as influenced by negative pressure wound therapy. Wounds.

[CR2] Booth MC, Atkuri RV, Nanda SK, Iandolo JJ, Gilmore MS (1995). Accessory gene regulator controls *Staphylococcus aureus* virulence in endophthalmitis. Invest Ophthalmol Vis Sci.

[CR3] Bubeck Wardenburg J, Patel RJ, Schneewind O (2007). Surface proteins and exotoxins are required for the pathogenesis of *Staphylococcus aureus* pneumonia. Infect Immun.

[CR4] Chavakis T, Hussain M, Kanse SM, Peters G, Bretzel RG, Flock JI, Herrmann M, Preissner KT (2002). *Staphylococcus aureus* extracellular adherence protein serves as anti-inflammatory factor by inhibiting the recruitment of host leukocytes. Nat Med.

[CR5] Davies CE, Hill KE, Newcombe RG, Stephens P, Wilson MJ, Harding KG, Thomas DW (2007). A prospective study of the microbiology of chronic venous leg ulcers to reevaluate the clinical predictive value of tissue biopsies and swabs. Wound Repair Regen.

[CR6] Dunman PM, Murphy E, Haney S, Palacios D, Tucker-Kellogg G, Wu S, Brown EL, Zagursky RJ, Shlaes D, Projan SJ (2001). Transcription profiling-based identification of *Staphylococcus aureus* genes regulated by the agr and/or sarA loci. J Bacteriol.

[CR7] Fleck TM, Fleck M, Moidl R, Czerny M, Koller R, Giovanoli P, Hiesmayer MJ, Zimpfer D, Wolner E, Grabenwoger M (2002). The vacuum-assisted closure system for the treatment of deep sternal wound infections after cardiac surgery. Ann Thorac Surg.

[CR8] Fleischmann W, Strecker W, Bombelli M, Kinzl L (1993). Vacuum sealing as treatment of soft tissue damage in open fractures. Unfallchirurg.

[CR9] Fleischmann W, Russ M, Westhauser A, Stampehl M (1998). Vacuum sealing as carrier system for controlled local drug administration in wound infection. Unfallchirurg.

[CR10] Foster TJ, McDevitt D (1994). Surface-associated proteins of *Staphylococcus aureus*: their possible roles in virulence. FEMS Microbiol Lett.

[CR11] George EA, Muir TW (2007). Molecular mechanisms of agr quorum sensing in virulent staphylococci. ChemBioChem.

[CR12] Gurjala AN, Geringer MR, Seth AK, Hong SJ, Smeltzer MS, Galiano RD, Leung KP, Mustoe TA (2011). Development of a novel, highly quantitative in vivo model for the study of biofilm-impaired cutaneous wound healing. Wound Repair Regen.

[CR13] Haggar A, Ehrnfelt C, Holgersson J, Flock JI (2004). The extracellular adherence protein from *Staphylococcus aureus* inhibits neutrophil binding to endothelial cells. Infect Immun.

[CR14] Hansen U, Hussain M, Villone D, Herrmann M, Robenek H, Peters G, Sinha B, Bruckner P (2006). The anchorless adhesin Eap (extracellular adherence protein) from *Staphylococcus aureus* selectively recognizes extracellular matrix aggregates but binds promiscuously to monomeric matrix macromolecules. Matrix Biol.

[CR15] Huntzinger E, Boisset S, Saveanu C, Benito Y, Geissmann T, Namane A, Lina G, Etienne J, Ehresmann B, Ehresmann C, Jacquier A, Vandenesch F, Romby P (2005). *Staphylococcus aureus* RNAIII and the endoribonuclease III coordinately regulate spa gene expression. EMBO J.

[CR16] Kreikemeyer B, McDevitt D, Podbielski A (2002). The role of the map protein in *Staphylococcus aureus* matrix protein and eukaryotic cell adherence. Int J Med Microbiol.

[CR17] Lalliss SJ, Stinner DJ, Waterman SM, Branstetter JG, Masini BD, Wenke JC (2010). Negative pressure wound therapy reduces pseudomonas wound contamination more than *Staphylococcus aureus*. J Orthop Trauma.

[CR18] Lindstedt S, Malmsjo M, Hansson J, Hlebowicz J, Ingemansson R (2012). Pressure transduction and fluid evacuation during conventional negative pressure wound therapy of the open abdomen and NPWT using a protective disc over the intestines. BMC Surg.

[CR19] Lowy FD (1998). *Staphylococcus aureus* infections. N Engl J Med.

[CR20] McGavin MH, Krajewska-Pietrasik D, Ryden C, Hook M (1993). Identification of a *Staphylococcus aureus* extracellular matrix-binding protein with broad specificity. Infect Immun.

[CR21] Moet GJ, Jones RN, Biedenbach DJ, Stilwell MG, Fritsche TR (2007). Contemporary causes of skin and soft tissue infections in North America, Latin America, and Europe: report from the SENTRY Antimicrobial Surveillance Program (1998-2004). Diagn Microbiol Infect Dis.

[CR22] Mooney JF, Argenta LC, Marks MW, Morykwas MJ, Defranzo AJ (2000). Treatment of soft tissue defects in pediatric patients using the V.A.C. system. Clin Orthop Relat Res.

[CR23] Morykwas MJ, Argenta LC (1997). Nonsurgical modalities to enhance healing and care of soft tissue wounds. J South Orthop Assoc.

[CR24] Moues CM, Vos MC, van den Bemd GJ, Stijnen T, Hovius SE (2004). Bacterial load in relation to vacuum-assisted closure wound therapy: a prospective randomized trial. Wound Repair Regen.

[CR25] Novick RP (2003). Autoinduction and signal transduction in the regulation of staphylococcal virulence. Mol Microbiol.

[CR26] Palma M, Haggar A, Flock JI (1999). Adherence of *Staphylococcus aureus* is enhanced by an endogenous secreted protein with broad binding activity. J Bacteriol.

[CR27] Pinocy J, Albes JM, Wicke C, Ruck P, Ziemer G (2003). Treatment of periprosthetic soft tissue infection of the groin following vascular surgical procedures by means of a polyvinyl alcohol-vacuum sponge system. Wound Repair Regen.

[CR28] Qiu J, Wang D, Xiang H, Feng H, Jiang Y, Xia L, Dong J, Lu J, Yu L, Deng X (2010). Subinhibitory concentrations of thymol reduce enterotoxins A and B and alpha-hemolysin production in *Staphylococcus aureus* isolates. PLoS ONE.

[CR34] Rothfork JM, Timmins GS, Harris MN, Chen X, Lusis AJ, OTTO M, Cheung AL, Gresham HD (2004). Inactivation of a bacterial virulence pheromone by phagocyte-derived oxidants: new role for the NADPH oxidase in host defense. Proc Natl Acad Sci USA.

[CR29] Song DH, Wu LC, Lohman RF, Gottlieb LJ, Franczyk M (2003). Vacuum assisted closure for the treatment of sternal wounds: the bridge between debridement and definitive closure. Plast Reconstr Surg.

[CR30] Uhlen M, Guss B, Nilsson B, Gatenbeck S, Philipson L, Lindberg M (1984). Complete sequence of the staphylococcal gene encoding protein A. A gene evolved through multiple duplications. J Biol Chem.

[CR31] Walev I, Martin E, Jonas D, Mohamadzadeh M, Muller-Klieser W, Kunz L, Bhakdi S (1993). Staphylococcal alpha-toxin kills human keratinocytes by permeabilizing the plasma membrane for monovalent ions. Infect Immun.

[CR32] Weed T, Ratliff C, Drake DB (2004). Quantifying bacterial bioburden during negative pressure wound therapy: does the wound VAC enhance bacterial clearance?. Ann Plast Surg.

[CR33] Wright JS, Jin R, Novick RP (2005). Transient interference with staphylococcal quorum sensing blocks abscess formation. Proc Natl Acad Sci USA.

